# 214. Routine Ophthalmologic Examination in *Klebsiella pneumoniae* Bacteremia Is Not Necessary: Incidence of and Risk Factors for Ocular Involvement

**DOI:** 10.1093/ofid/ofad500.287

**Published:** 2023-11-27

**Authors:** So Yun Lim, Hye Ji Kwon, Yun Woo Lee, Heungsup Sung, Mi-Na Kim, Eui Jin Chang, Seongman Bae, Jiwon Jung, Min Jae Kim, Sung-Han Kim, Sang-Ho Choi, Sang-Oh Lee, Yang Soo Kim, Joo Yong Lee, Yong Pil Chong

**Affiliations:** National Medical Center , Seoul, Seoul-t'ukpyolsi, Republic of Korea; Asan Medical Center, Seoul, Seoul-t'ukpyolsi, Republic of Korea; Asan Medical Center, Seoul, Seoul-t'ukpyolsi, Republic of Korea; Asan Medical Center, Seoul, Seoul-t'ukpyolsi, Republic of Korea; Asan Medical Center, Seoul, Seoul-t'ukpyolsi, Republic of Korea; Department of Internal Medicine, Asan Medical Center, Seoul, Korea, Seoul, Seoul-t'ukpyolsi, Republic of Korea; Asan Meidical Center, Songpa-gu, Seoul-t'ukpyolsi, Republic of Korea; Asan Medical Center, Seoul, Seoul-t'ukpyolsi, Republic of Korea; Asan Medical Center, Seoul, Seoul-t'ukpyolsi, Republic of Korea; Asan medical center, Seoul, Seoul-t'ukpyolsi, Republic of Korea; Asan Medical Center, Seoul, Seoul-t'ukpyolsi, Republic of Korea; Asan Medical Center, Seoul, Seoul-t'ukpyolsi, Republic of Korea; Asan Medical Center, Seoul, Seoul-t'ukpyolsi, Republic of Korea; Asan Medical Center, Seoul, Seoul-t'ukpyolsi, Republic of Korea; Asan Medical Center, Seoul, Seoul-t'ukpyolsi, Republic of Korea

## Abstract

**Background:**

*Klebsiella pneumoniae* bacteremia can result in severe clinical manifestations including metastatic infection, which is not uncommon in Asia. However, there are limited data on the incidence and risk factors for ocular involvement in *K. pneumoniae* bacteremia.

**Methods:**

We retrospectively reviewed the medical records of all patients with *K. pneumoniae* bacteremia who underwent ophthalmologic examination in a tertiary center in Seoul, Korea, from February 2012 to December 2020. Two retinal specialists reviewed the findings of the ophthalmologic examinations and classified them as endophthalmitis, chorioretinitis, and no ocular involvement.

**Results:**

Of 689 patients, 56 (8.1%; 95% CI 6.2-10.4) had ocular involvement, and 9 (1.3%; 95% CI 0.6-2.5) were diagnosed with endophthalmitis. Of 47 patients with chorioretinitis, 45 (95.7%) improved with systemic antibiotic therapy alone. Community-onset bacteremia (100% vs 62.1% vs 57.4%, *P*=0.04), cryptogenic liver abscess (55.6% vs 11.8% vs 8.5%, *P*=0.003), and metastatic infection (66.7% vs 5.8% vs 10.6%, *P* < 0.001) were more common in endophthalmitis than in no ocular involvement or chorioretinitis. In multivariable analysis, cryptogenic liver abscess (aOR, 6.63; 95% CI, 1.44-35.20) and metastatic infection (aOR, 17.52; 95% CI, 3.69-96.93) were independent risk factors for endophthalmitis. Endophthalmitis was not associated with 30-day mortality.

**Table 1.**
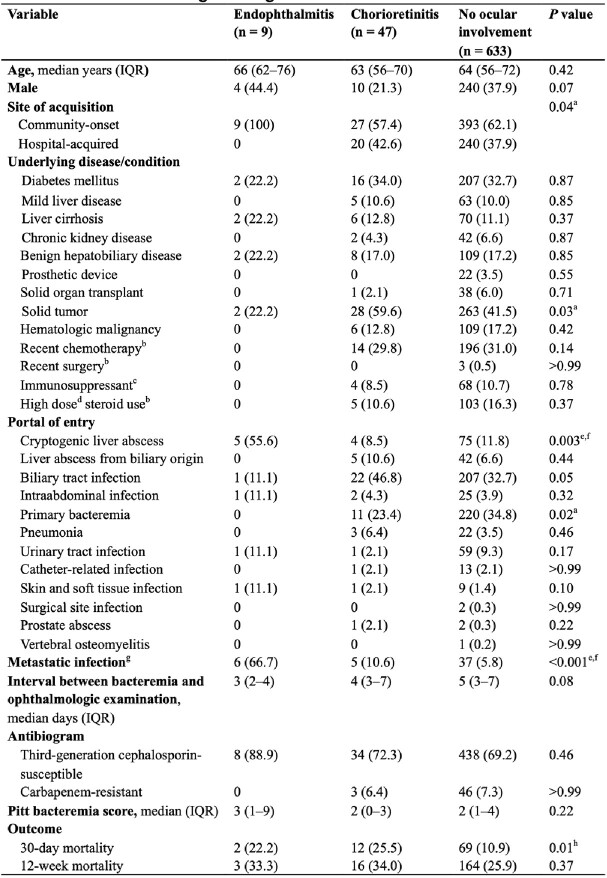
Demographics, clinical characteristics and outcomes of patients with K. pneumoniae bacteremia according to degree of ocular involvement

Data presents n (%) unless indicated otherwise. aNo significant difference (P>0.017) between any of the pairs in post hoc analysis. bWithin 1 month. cDefined as one of the following conditions: (i) daily receipt of immunosuppressants including corticosteroids, (ii) human immunodeficiency virus infection, (iii) solid organ or hematopoietic stem cell transplant recipients, (iv) receipt of chemotherapy for underlying malignancy during the previous 6 months, and (v) underlying immune deficiency disorder. dDose equivalent to ≥20mg/day of prednisone for two weeks or longer. eSignificantly different (P <0.017) between endophthalmitis and no ocular involvement. fSignificantly different (P <0.017) between endophthalmitis and chorioretinitis. gMetastatic infection other than ocular involvement. hSignificantly different (P <0.017) between chorioretinitis and no ocular involvement.

**Table 2.**
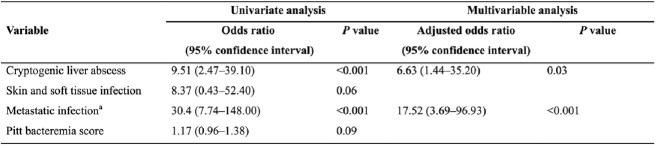
Risk factors for endophthalmitis in patients with K. pneumoniae bacteremia aMetastatic infection other than ocular involvement.

**Table 3.**
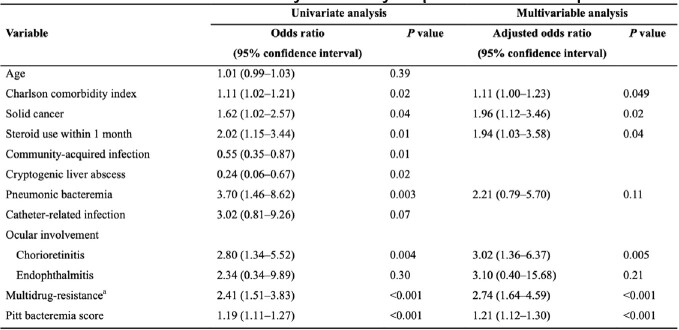
Risk factors for 30-day mortality in patients with K. pneumoniae bacteremia aThird-generation cephalosporin-resistant or carbapenem-resistant

**Conclusion:**

Endophthalmitis is rare in Asian patients with *K. pneumoniae* bacteremia. Targeted ophthalmologic examination in those with cryptogenic liver abscess, multiple metastatic infections, or ocular symptoms may be more appropriate than routine examination of all patients.

**Figure:**
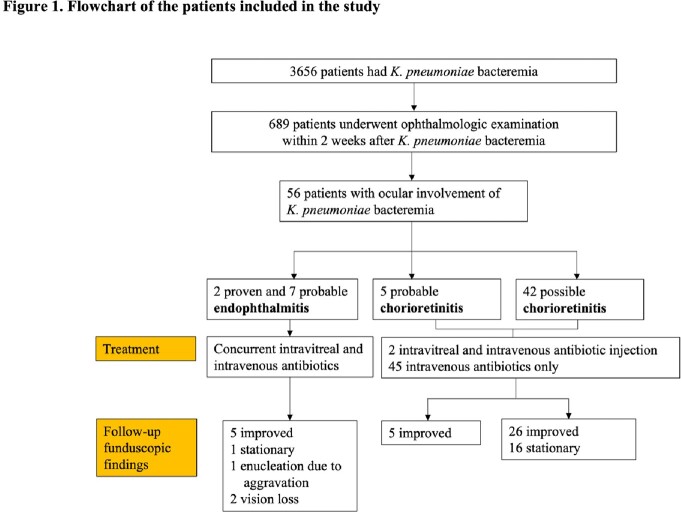

**Disclosures:**

**All Authors**: No reported disclosures

